# Assessing parents’ awareness about children’s “first thousand days of life”: a descriptive and analytical study

**DOI:** 10.1186/s13690-021-00673-6

**Published:** 2021-08-28

**Authors:** Fatemeh Bagheri, Nouzar Nakhaee, Yones Jahani, Reza Khajouei

**Affiliations:** 1 Department of Health Information Sciences, Faculty of Management and Medical Information Sciences, Kerman University of Medical Sciences, Kerman, Iran; 2 Neuroscience Research Center, Kerman University of Medical Sciences, Kerman, Iran; 3 Department of Biostatistics and Epidemiology, School of Public Health, Kerman University of Medical Sciences, Kerman, Iran

**Keywords:** First thousand days of life, Awareness, Parents, Child health

## Abstract

**Background:**

Many adulthood illnesses are rooted in childhood, especially in the “first thousand days of life”. Despite parents’ role in children’s development, no study has examined parental awareness concerning this period. This study aimed to examine the awareness of parents concerning the “first thousand days of life” and the relationship between parents’ demographics and their awareness.

**Methods:**

This study was conducted on 135 parents in Kerman, Iran, using a valid and reliable questionnaire developed by researchers based on the opinion of experts and relevant references. The relationship between participants’ demographics and their awareness was examined by multiple regression. The relationship between homogeneity of couples’ education degree and awareness was analyzed using ANOVA. Chi-square was used to examine the relationship between information sources and parents’ familiarity and to compare parents’ preferred sources.

**Results:**

The average parental awareness was 41.96 ± 11.90. Eighty-three percent of the parents have not heard about the “first thousand days of life”. The source of information for 57% of the parents was friends and relatives (*p* < 0.0001). Overall, 87% of the parents desired to know about this period, and 47% liked using mobile applications for information (*p* < 0.0001).

**Conclusions:**

Parents’ awareness about the “first thousand days of life” is lower than the average. Since the source of information concerning this period for most parents is friends and relatives and most parents are very interested in obtaining information, it is recommended that policy-makers use the capacity of other sources to increase parents’ awareness. Given the greater tendency of parents to obtain information through mobile applications, we suggest investing more in this source.

**Supplementary Information:**

The online version contains supplementary material available at 10.1186/s13690-021-00673-6.

## Background

According to the World Health Organization, many physical illnesses and behavioral problems in adulthood have their roots in childhood, especially in the “first thousand days” [1]. The “first thousand days of life” includes nine months of intrauterine life (270 days) and the first two years of a child’s life (730 days). It is the most affected and vulnerable period in the physical and cognitive development of each individual due to their rapid growth and development, high nutritional needs, susceptibility to infection, and complete dependence on others for treatment, nutrition, and social interaction [2, 3]. All the events that occur in these thousand days for the fetuses or children could influence some crucial aspects of their lives including their adulthood health. Research has shown that issues such as malnutrition [2] and emotional adversity [4] including parental divorce and marital conflict [5], rejection [6], unreasonable parental controls [7], delinquent behavior [8], emotional deficiency [9] may contribute to social [7, 10] and behavioral problems [10], as well as mental [7, 11] and physical [11–13] disorders such as cancer [14], cardiovascular and musculoskeletal disease [15] and premature death in adulthood [16, 17].

The importance of the “first thousand days of life” in children’s development and health was first raised in 2008 by reporting the relationship between nutrition and child health [18] and then emerged in 2010 officially. The term “1000 days” was probably first used in 2010 [19]. Since the “first thousand days” of life is an opportunity for human development and the best time to invest in improving health, future prosperity, and the boost of countries’ economies by having a healthier community, it is referred to as a golden period [18] and has recently become one of the public health priorities for policymakers [20]. Therefore, many research projects in scientific and human societies such as UNICEF (United Nations Children’s Emergency Fund) have focused on the theory of the “first thousand days of life” [21] for improving health.

Parents are the first teachers and important influencers on a child health and development [22]. Since the most important period in a child’s development is the “first thousand days of life”, parents’ attention to this period can have a positive effect on children’s development. This attention is especially essential for the parents of children who have not passed the first thousand days or the parents that will face these thousand days in the future.

Various studies [22–25] have emphasized the effect of increasing parents’ awareness, knowledge, and attitude about the “first thousand days of life” on parenting style and child’s development and health. To our knowledge, so far, no study has been conducted to determine the level of parents’ awareness about the “first thousand days of life”. The previous studies have been done on other aspects of the “first thousand days of life”. A study [26] examined parents’ knowledge about the concept of the early development of children and showed that the knowledge of Iranian parents about child development is insufficient for parenting. Several studies [27–29] have addressed the effect of educational interventions on parents’ knowledge and awareness about parenting. For example, a review study [30] in 2017 showed that the implementation of childhood-related interventions positively affects parenting and child development. Understanding the parental awareness about the “first thousand days of life”, especially based on demographic information, can help to improve the awareness of parents about this period and children development and health. Therefore, this study examined the awareness of parents of children who are in the “first thousand days of life”, or will have children soon, about the “first thousand days of life”. Moreover, we aimed to study the relationship between demographic characteristics of the parents and their awareness.

## Methods

### Study design

In this study, based on the following formula [31], a sample size of 96 was calculated.

$$ {\displaystyle \begin{array}{c}n=\frac{{\left({\mathrm{z}}_{1-\upalpha /2}\right)}^2\ast {\left(\upsigma \right)}^2}{{\left(\mathrm{d}\right)}^2}\\ {}\left(\upalpha =0.05,{\mathrm{z}}_{1-\upalpha /2}=1.96,\upsigma =25,\mathrm{d}=5\right)\end{array}} $$

In this formula; n = sample size, α = the alpha level (the level of risk the researcher is willing to take that true margin of error may exceed the acceptable margin of error), z = value for selected alpha level, σ = estimate of standard deviation, and d = acceptable margin of error for mean being estimated. To compensate for the decrease in study sample due to the withdrawal of incomplete questionnaires and to enhance the accuracy of the results [32], about 40% was added to the sample size and 135 individuals were invited to participate.

**Fig. 1:**
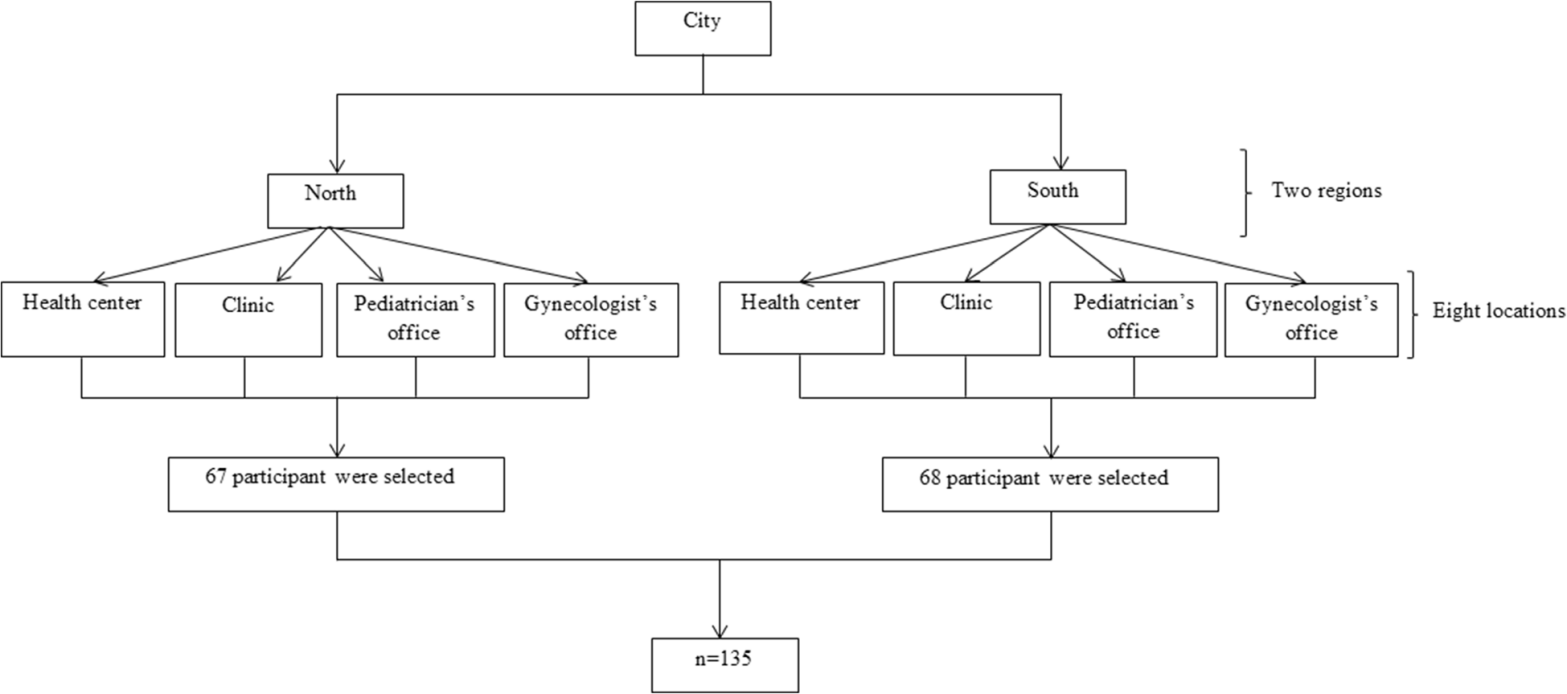
The flowchart of study design

Figure 1 shows the flowchart of the study design. This study was conducted in Kerman (the most populous city in southeastern Iran). To reach maximum geographical variation, the city was divided into two north and south regions. To choose the place of data collection, four of the locations frequently visited by the participants (a health center, a clinic, a pediatrician’s office, and a gynecologist’s office) were randomly selected from each region. In total, eight locations were selected for sampling. The following two criteria were used to select the study participants among the people who visited these locations, 1) having a child under the age of two, being pregnant, or intending to have a child in the future, 2) voluntary participation. For the random selection of individuals, one of the researchers visited the designated locations two times. She collected data from ten random participants in each center during the first visits and from seven participants during the second visits until the sample size was met.

### Data collection

Data were collected using a questionnaire developed based on the opinion of experts, relevant literature [33], and the 1000 days website [19]. A social medicine specialist, a gynecologist, a pediatrician, and a medical informatics specialist confirmed the face and content validity of this questionnaire. Its reliability was also confirmed by calculating the internal correlation with Cronbach’s alpha on a sample of 30 people. These people were selected from the same setting as the study participants (r = 0.6). The questionnaire consisted of four sections. 1) Demographic information of participants, including age, sex, education level, and parenting, pregnancy, or intention to conceive (seven questions). 2) Questions to assess parents’ awareness of their children’s “first thousand days of life”. This section includes questions about the period of the “first thousand days of life” and its importance (eight questions), child nutrition and food supplements (four questions), the role of parents and their socio-economic status in the “first thousand days of life,” (six questions) and the use of digital media by children (two questions). This section contained multiple-choice questions with five options so that only one of the options was the correct answer. 3) A question to determine the level of familiarity with the phrase “first thousand days of life” with a five-point Likert scale from “not at all” to “very high” and a multiple-choice question to determine the source of familiarity. 4) A question to assess the degree of parents’ tendency to get acquainted with the “first thousand days of life” on a five-point Likert scale from “not at all” to “very high”; and a multiple-choice question to determine the preferred source of information to get acquainted with the “first thousand days of life”. ‘An additional file shows this in more detail (see Additional file 1)’. All the questions of the second part and the first question of the third part were categorized as follows according to the context of each question: “thousand days and its importance”, “importance of nutrition and food supplements”, “use of digital media by children”, and “role of parents and the importance of the family socio-economic status in the first thousand days of life”.

A paper questionnaire in Persian was used to collect data. One of the researchers personally distributed the questionnaires among the participants. She was present at the study locations to help people who could not fill out the questionnaire. The participants who were selected but refused to participate were replaced by others. To fill out the questionnaire, participants were allowed to choose only one option from the answers provided to each question.

### Data analysis

Data were analyzed using SPSS (Statistical Package for the Social Sciences) 21. Before analyzing the data, the multiple imputation method was used to cover the missing data. Thus, the answer to each of the unanswered questions was estimated according to the answers that the person had given to the other questions. Since the average age of conception in the area (26.61 ± 4.71) is less than 30 years [34]. Thus, 30 years was chosen as the limit for categorizing the participants in terms of age. To score the items in the second section, 100 points were given to each correct answer and zero points to each incorrect answer. To analyze the five-choice question of the third and fourth sections, a score of zero to 100 was used (not at all = 0, only once = 25, once to several times = 50, high = 75, very high = 100). If a participant had selected more than one answer per question, a zero point was given to that question. Parents’ awareness was considered in the range of zero to 100 scores. The average score of the questions of the second part and the first question of the third part was used to calculate the general awareness score of individuals. To calculate their awareness score in each category, the average score of the questions of that category was used. To analyze the awareness scores, two awareness levels of less than average (score below 50) and moderate or higher (score 50 and above) were determined. Statistical tests (Multiple regression, Analysis of Variance (ANOVA), and Chi-square) were used for the random error management, and a *p* < 0.05 was considered statistically significant in this study. The Multiple regression test was used to investigate the relationship between parents’ awareness and their demographic characteristics. This test was also used to examine the relationship between parents’ awareness concerning categories of “first thousand days of life” and their demographic characteristics. ANOVA was used to examine the relationship between the homogeneity of couples’ education degree and their awareness. Chi-square was also used to examine the relationship between information sources and parents’ awareness and to compare parents’ favorite sources of information.

## Results

The demographic information of the participants is shown in Table 1. The age of the participants ranged from 19 to 48, with a mean of 29.81 ± 5.24. Fifty percent of the participants were younger than 30, and 83% of them were women. Most of the participants (71%) and their spouses (65%) had an academic degree. Approximately half of the participants (48%) had a child under the age of two and the other half (52%) were expecting or planning to have a child. The majority of the participants (83%) stated that they had never heard the phrase “the first thousand days of life.” Eighty-seven percent of the participants wanted more information concerning this period.

**Table 1:** Demographic information of the participants

Demographic Information	n (%)
**Age**	
<30	67(50.4)
>=30	68(49.6)
**Gender**	
Female	112(83)
Male	23(17)
**Education**	
Under high school diploma	10(7.4)
High school diploma	29(21.5)
Academic	96(71.1)
**Spouse**'**s education**	
Under high school diploma	10(7.4)
High school diploma	37(27.4)
Academic	88(65.2)
**Parents status**	
Having a child under the age of two	65(48.1)
Expecting a baby	43(31.9)
Actively trying to have a child	27(20)
**Acquaintance with the** "**first thousand days of life**"	
Never heard	112(83)
Heard once	7(5.2)
Heard a few times	11(8.1)
Heard many times	2(1.5)
Heard too many times	3(2.2)
**Tendency to get acquainted with the "first thousand days of life"**	
Not at all	2(1.5)
Little	6(4.4)
Much	38(28.1)
Too much	74(54.8)
No opinion	15(11.1)

The mean score of parents’ awareness about the “first thousand days of life” was 41.96 ± 11.90 out of 100. Seventy-three percent (*n* = 98) of the participants had a level of awareness lower than 50. Table 2 presents the results of the multiple regression test about the relationship between the parent’s awareness of the “first thousand days of life” and the demographic characteristics of the participants. There was a significant relationship between spouse’s education and individual’s awareness (*p* = 0.01). The awareness score of the parents whose spouses had a high school diploma was 5.83 points lower than the score of parents whose spouses had academic degrees.

**Table 2:** Relationship between the parents’ demographics and their awareness of the “first thousand days of life”

Variable	Mean ± SD	Regression Coefficients (95% CI)	p
**Age**			
< 30	38.64 ± 13.73	-1.89(-6.08, 2.29)	0.37
>= 30	40.52 ± 14.20	Ref	–
**Gender**			
Female	39.94 ± 10.98	0.70(-4.69, 6.10)	0.79
Male	39.24 ± 18.35	Ref	–
**Education**			
Under high school diploma	42.05 ± 24.92	2.30(-6.59, 11.20)	0.61
High school diploma	36.96 ± 19.29	-2.78(-8.04, 2.47)	0.29
Academic	39.75 ± 14.13	Ref	–
**Spouse**'**s education**			
Under high school diploma	36.37 ± 25.39	-7.74(-16.25, 0.85)	0.07
High school diploma^*^	38.28 ± 14.87	-5.83(-10.65, -1.00)	0.01
Academic	44.12 ± 15.07	Ref	–
**Parents’ status**			
Having a child under the age of two	39.53 ± 13.53	0.41(-4.84, 5.68)	0.87
Expecting a baby	40.13±16.14	1.02(-4.69, 6.74)	0.72
Actively trying to have a child	39.11±17.55	Ref	–

*Notes*: * Indicate *p* < 0.05, *Ref* Indicate Reference, *SD* Standard Deviation, *CI* Confidence Intervals

**Fig. 2:**
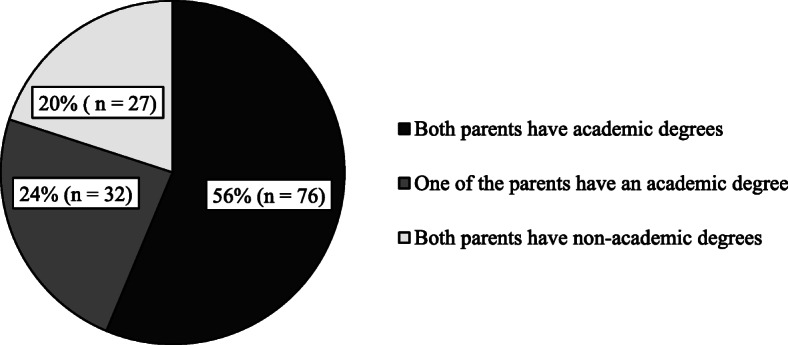
Homogeneity of the education level of the parents and their spouses

Figure 2 shows the homogeneity of the education degree of the parents and their spouses. Fifty-six percent of the parents and their spouses (*n* = 76) both had academic degrees. According to the results of ANOVA, there was no significant relationship between the homogeneity of the couples’ education degree and their awareness about the “first thousand days of life” (*p* > 0.05).

There was a significant relationship between different information sources and the acquaintance of parents with the “first thousand days of life” (*p* < 0.0001). Of the 23 participants who were familiar with the “first thousand days of life”, 57% had obtained information from friends and relatives, 18% from healthcare staff, 13% through cyberspace, 9% through books and magazines, and 3% through other sources.

Eighty-seven percent (*n* = 103) of the 118 people who wanted more information about the “first thousand days of life” also determined their preferred source of information. Table 3 shows the results of the Chi-Square test to compare the tendency of these people for using different sources for information. There was a significant difference in people’s tendency to use different information sources (*p* < 0.0001). The participants’ tendency to use media (10.16%), books and magazines (15.25%), and healthcare staff (15.25%) to get acquainted with the “first thousand days of life” was significantly less than 50%. About 47 % (*n* = 55) of these people expressed that they want to get information through mobile applications.

**Table 3:** Comparing the preferred sources of information for acquaintance with the “first thousand days of life”

Source	Tendency n (%)	Chi-Square	*p*
Mobile applications			
Yes	55(46.61)	0.54	0.46
No	63(53.38)		
Media			
Yes	12(10.16)	74.88	< 0.0001
No	106(89.83)		
Books and Magazines			
Yes	18(15.25)	56.98	< 0.0001
No	100(84.74)		
Healthcare staff			
Yes	18(15.25)	56.98	< 0.0001
No	100(84.74)		

Analysis of awareness concerning categories related to the “first thousand days of life” showed that the level of awareness about three categories was below 50. The level of awareness for the categories “period of the first thousand days and its importance”, “Importance of nutrition and food supplements” and “use of digital media by children” was below 36 out of 100 and for the category “role of parents in the first thousand days of life and the importance of the socio-economic status of the family” was 68.02 ± 17.87.

The results of the multiple regression test about the relationship between parents’ demographics and their awareness concerning categories of “first thousand days of life” are shown in Table 4. There was a significant relationship between parent and spouse education with awareness level in some categories (*p* < 0.05). Nevertheless, the awareness score of the parents with a high school diploma, about the “period of the first thousand days and its importance”, was 10.26 points lower than the scores of parents with an academic degree. The awareness score about the “period of the first thousand days and its importance” in parents whose spouses had a high school diploma or a lower degree was 8.64 and 11.84 points lower than parents whose spouses had an academic degree, respectively. The awareness score of parents with a high school diploma and with a lower degree concerning the “use of digital media by children” category was 17.45 and 28.34 points higher than the parents who had academic degrees, respectively. Parents’ age was significantly associated with the level of awareness about the “importance of nutrition and food supplements” (*p* < 0.05). The awareness score about the “importance of nutrition and food supplements” in parents under 30 years of age was 8.22 points lower than parents aged 30 and older.

**Table 4:** Relationship between parents’ demographics and their awareness concerning categories of “first thousand days of life”

Variable	Mean ± SD			
	Period of the first thousand days and its importance	Importance of nutrition and food supplements	Role of parents in the first thousand days of life and the importance of the socio-economic status of the family	Use of digital media by children
**Age**				
< 30	27.23 ± 22.67	22.80 ± 33.06 ^*^	70.46 ± 26.11	39.13 ± 51.15
>= 30 ^Ref^	24.95 ± 23.58	31.03 ± 34.38	68.26 ± 35.12	49.32 ± 53.18
**Gender**				
Female	26.42 ± 23.49	30.69 ± 34.28	67.95 ± 27.09	41.82 ± 53.02
Male ^Ref^	25.75 ± 17.74	23.13 ± 25.84	70.78 ± 20.43	46.63 ± 39.99
**Education**				
Under high school diploma	29.96 ± 15.87	24.14 ± 23.17	70.16 ± 18.27	57.30 ± 35.79^*^
High school diploma	19.02 ± 20.94^*^	25.54 ± 30.53	69.05 ± 25.95	46.41 ± 47.22^*^
Academic ^Ref^	29.28 ± 27.92	31.05 ± 40.75	68.87 ± 32.23	28.96 ± 63.09
**Spouse**'**s education**				
Under high school diploma	21.08 ± 16.22^*^	22.62 ± 23.65	69.01 ± 18.68	44.73 ± 36.55
High school diploma	24.27 ± 18.24^*^	26.57 ± 26.58	67.85 ± 22.98	40.25 ± 41.11
Academic ^Ref^	32.92 ± 28.51	31.55 ± 41.65	71.23 ± 32.92	47.70 ± 64.35
**Parents status**				
Having a child under the age of two	24.74 ± 22.09	30.44 ± 23.16	66.93 ± 25.39	47.53 ± 49.74
Expecting a baby	27.28 ± 23.37	28.72 ± 31.14	68.19 ± 24.59	45.49 ± 48.13
Actively trying to have a child ^Ref^	26.24 ± 18.39	21.58 ± 26.86	72.96 ± 21.20	39.65 ± 41.51

*Notes*: * indicate *p* < 0.05, *Ref* Indicate Reference, *SD* Standard Deviation

## Discussion

### Principal finding

In this study, the mean parental awareness of the “first thousand days of life” was below the average and only the awareness of less than a third of the parents was above average. Besides, the level of the parent’s awareness concerning the “period of the first thousand days and its importance”, the “importance of nutrition and food supplements”, and the “use of digital media by children” was below the average. However, their level of awareness about the “role of parents in the first thousand days of life and the importance of the socio-economic status of the family” was higher than the other categories. In the present study, among the demographic variables, the level of spouse’s education was directly related to the individual’s general awareness of the “first thousand days of life”. Also, the education of parents and their spouses was directly related to awareness related to the category of “period of the first thousand days and its importance”, but the education of the individual was inversely related to the awareness concerning the “use of digital media by children”. According to the findings, the homogeneity of couples’ education had no significant relationship with their level of awareness concerning the “first thousand days of life”. Age was also correlated with the level of awareness concerning the “importance of nutrition and food supplements”. The source of information for most parents about the “first thousand days of life” was friends and relatives. In this study, most parents wanted to increase their awareness of the “first thousand days of life” and use mobile apps to get information concerning this period.

Most participants in this study had not heard the phrase “the first thousand days of life”. The mean parental awareness concerning the “first thousand days of life” was below the average, and only the awareness of about a quarter of the parents was above the average. This study showed that the awareness of the parents about one of the aspects of the children’s lives was below the average. This result is in line with the findings of the other studies showing that parent’s awareness about other aspects of children’s lives, such as the oral health of children [35] and key essential nutrition [23]is also low. Based on our results, the academic education of spouses is related to the individual’s awareness of the “first thousand days of life”. Parents who, themselves or their spouses, have an academic degree are also more aware of the “first thousand days and its importance”. This result showed that awareness of parents with higher education degrees concerning the “first thousand days and the effect this period on children’s development” is higher than the awareness of other parents. This result is consistent with the findings of previous studies that showed the awareness of educated parents, especially mothers [30], and knowledge of professionals [36] with higher education about early childhood development are higher. On the contrary, this study showed that the education degree has an inverse relationship with the awareness concerning the “use of digital media by children”. This result is inconsistent with the findings of a previous study [37] that showed educated parents are more aware of the effects of the media on children, thus they have a higher tendency to control their children’s access and use of the media. It seems that due to the lack of information sources, even educated people in the present study are unaware of the disadvantages of using digital media by children.

The results of this study indicated that among the demographic information, age is associated with parents’ level of awareness concerning the “importance of nutrition and food supplements”. Parents over the age of 30 have a higher level of awareness about one of the aspects of parenting than the younger parents. This result confirms the findings of a previous study [38], which showed that the more the age increases the more information a person receives resulting in the improvement of the level of mothers’ awareness about parenting. Age is one of the factors that indicate the physical, psychological, and social maturity of a person. It seems that a rise in age can also increase the duration of the exposure to information and the amount of information a person is exposed to; thus, it can increase a person’s experiences like parent’s experiences concerning the child health and development reported in this study.

In the present study, most parents preferred to increase their awareness about the “first thousand days of life” as an important period in the child health. This result is in line with the results of previous studies [9, 39] reporting parents’ interest in obtaining information related to child health. In this study, most parents liked to use mobile apps, among different media, to learn about the “first thousand days of life”. Baidal [25] also showed in a study that people like to use mobile applications to increase awareness concerning children’s health.

This study had three limitations. First, fathers had a lower chance to participate in this study compared to mothers. Although this result is consistent with the results of other studies on the limited participation of fathers in child-rearing and parenting [40], the limited participation of fathers can be due to several other factors. Due to the simultaneity of the working hours of health centers and most fathers, especially in the morning shifts, fathers were less present than mothers in the health centers. Prohibition of fathers’ presence in obstetrics and gynecology offices, higher job involvement of fathers compared to mothers, and unwillingness to participate in the study can also be other factors that resulted in the limited participation of fathers. Future studies can encourage fathers’ participation by different incentives and using virtual platforms. Second, this study showed that older parents have a higher level of awareness concerning the “first thousand days of life” than younger parents. Since older parents have a higher chance of having more children over time, they could be more exposed to child-related information, and thus are more likely to be better informed about child health. In this study, information about the number of children was not collected to examine the relationship between high parental information at older ages and the number of children. Further studies can focus on the relationship between parental awareness of the “first thousand days of life” and the number of children. Third, since this study has been conducted in the capital city of a province, its results should be used with caution for other geographical areas. However, it seems that the demographic composition of parents in terms of influential demographic variables is identical in all major and capital cities [41]. Moreover, to our knowledge, this is the first study that developed a tool to assess the awareness of different groups of parents about the “first thousand days of life” and investigated the parental awareness concerning different categories related to the “first thousand days of life”.

### Implications of the study

According to studies [23, 28], a change in the attitude and knowledge of parents can have a positive effect on parenting. Attitude is influenced by education degree, training level, and experience [42]. According to the finding of the present study, although many parents have an academic education, their awareness of the “first thousand days of life” is lower than the average. In addition, in this study, the sources of information about the “first thousand days of life” for most parents are friends and relatives. It seems that due to the lack of information sources and corresponding training, even educated people are unaware of the opportunity of the first thousand days of their children. Based on the results of a study [24], parents need educational interventions on different aspects of the “first thousand days of life” and its impact on the children’s development and health in adulthood. Therefore, it is suggested that health officials and policy-makers use the capacity of information sources such as media, books, cyberspace, and healthcare staff to improve awareness of the society about the “first thousand days of life”. Given that rise in age has also been associated with increasing awareness concerning the “first thousand days of life” in this study, it is suggested that information about this period is provided to people at a younger age and from the beginning of the marriage.

This study showed that most parents like to be acquainted with the “first thousand days of life”. In addition, among different information sources, they mostly tended to use mobile apps to raise awareness about this period of life. Research shows that mobile phones can effectively enhance learning in most fields [43]. Mobile phones can provide access to the best, most useful, and most relevant and attractive content without time, location, and speed constraints [44]. Given the desire of most participants to use mobile applications about the “first thousand days of life”, it seems that running mobile-based interventions will increase parental awareness concerning this period of life and its effects in the long term.

## Conclusions

The results of this study showed that parents’ awareness about the “first thousand days of life” is lower than the average. Among the demographic variables, only the spouse’s education affects the parents’ awareness concerning this period of life. The source of information for most parents about the “first thousand days of life” is friends and relatives, and most parents are very interested in learning about this period. Therefore, it is suggested that besides planning to educate parents, policy-makers use the capacity of various information resources to improve awareness concerning the “first thousand days of life”. Due to the greater desire of parents to use mobile applications about the “first thousand days of life”, it is recommended to use this platform more than other media to raise awareness concerning this period.

## Supplementary Information

**Additional file 1.** Parents’ Awareness Questionnaire about Children’s “First Thousand Days of Life”. The additional file is a questionnaire to assess parents’ awareness about children’s “first thousand days of life”.

## Data Availability

The datasets used and/or analyzed during the current study are available from the corresponding author on reasonable request.

## Abbreviations

UNICEF: United Nations Children’s Emergency Fund
SPSS: Statistical Package for the Social Sciences
ANOVA: Analysis of Variance
SD: Standard Deviation
CI: Confidence Intervals
