# Supplementary material for: Assessing parents’ awareness about children’s “first thousand days of life”: a descriptive and analytical study
Source: Arch Public Health. 2021 Aug 28;79:154. doi: 10.1186/s13690-021-00673-6 (PMC8400762; doi:10.1186/s13690-021-00673-6)
Supplement: Supplementary file 1 — Additional file 1. Parents’ Awareness Questionnaire about Children’s “First Thousand Days of Life”. The additional file is a questionnaire to assess parents’ awareness about children’s “first thousand days of life”. [file 13690_2021_673_MOESM1_ESM.docx]

**Additional file 1**

Dear Parent:

The following questionnaire is to assess parents' awareness about children's "first thousand days of life". The information collected in this questionnaire is only used for research purposes and will be analyzed and reported collectively. Please answer the questions honestly and carefully.

**Section1 (8 questions)**

1. Sex: 🗆male 🗆female
2. Age……………
3. Education 🗆Under high-school diploma 🗆 High-school diploma 🗆 Academic
4. Spouse education 🗆Under high-school diploma 🗆 High-school diploma 🗆 Academic
5. Do you have a child under the age of two? 🗆Yes 🗆No
6. If you do not have a child under the age of two, are you currently waiting for the birth of your child? 🗆Yes 🗆No
7. If your answers to the previous two questions are negative; do you plan to have children in the future? 🗆Yes 🗆No

**Section 2 (20 questions)**

1. Do you know to which period of life the "first 1000 days of life" is referred to?

🗆 During nine months of intrauterine life

🗆The first two years of a child's life

🗆The first three years of a child's life

🗆 During nine months of intrauterine life and the first two years of a child's life

🗆I do not know

1. In your opinion, the events of which period affect a person's future life the most and its problems cause many physical illnesses and behavioral problems in adulthood?

🗆 During emberyonic period

🗆 During emberyonic period and childhood

🗆 During childhood

🗆 During childhood and adolescence

🗆I do not know

1. Is there a connection between breastfeeding, and physical and mental illnesses in adulthood?

🗆There is no connection.

🗆There is little connection.

🗆There is a high connection.

🗆There is a very high connection.

🗆I do not know

1. How are the best parents?

🗆Easygoing and kind parents with no supervision over their children

🗆Strict parents with high expectations

🗆Careless parents without any supervision over their children

🗆Authoritative yet kind parents who have control and supervision over their children

🗆Authoritative yet easygoing parents

1. What is the role of parents in the "first 1000 days of life"?

🗆The role of none of them matters in this period.

🗆Only the role of the mother is vital in this period.

🗆The role of the father is more important than the role of the mother in this period.

🗆The roles of both parents are identically important in this period.

🗆Both play an important role in this period, but the role of the mother is a little more prominent.

1. What is the relationship between the socio-economic status of families and the mental health of children in adulthood?

🗆The socio-economic status of families has no association with health in adulthood.

🗆The socio-economic status of families has quite an association with health in adulthood.

🗆Only the social status of the family is associated with the mental health of children in adulthood.

🗆Only the economic status of the family is associated with the mental health of children in adulthood.

🗆I do not know

1. Are under 2 years old children allowed to use digital media such as mobile, computers, and television?

🗆They are not allowed to use it at all.

🗆They can use digital media limitedly and under parental supervision.

🗆There is no prohibition, but it is better not to use it.

🗆It is very informative and useful and is recommended.

🗆I do not know

1. What is the effect of using digital media on children under the age of five?

🗆It has very negative effects and is prohibited.

🗆It can have bad effects.

🗆It has no effect.

🗆It can have good effects.

🗆It is very informative and useful and is recommended.

1. During which period is breastfeeding recommended?

🗆It is not necessary and can be replaced with milk powder.

🗆There is no specific period.

🗆Until six months

🗆Until two years

🗆Exclusive use up to six months is necessary and is recommended, however, it is better to continue until the end of two years.

1. When does a child become interested in high-fat and salty foods?

🗆From the school-age and it is more influenced by friends.

🗆From birth to two years old

🗆During the emberyonic and prenatal period

🗆It has no specific time and is different for each person.

🗆I do not know

1. Which of the following does not increase the risk of obesity in adulthood?

🗆 Maternal weight during pregnancy

🗆Maternal blood sugar during pregnancy

🗆Insomnia of the baby in the first year of life

🗆 Sleeping much of the baby in the first year of life

🗆Lack of breastfeeding in the first year of life

1. Which of the following affects the child and his future life in adulthood?

🗆Mental health, emotional status, and the attachment style of the family

🗆Nutrition

🗆Poverty and social factors

🗆All items

🗆I do not know

1. When is it time to use iron drops as a supplement for a child?

🗆From the beginning of birth to the beginning of six months

🗆From the beginning of age six months to the end of one year

🗆From the end of age six months to the end of two year

🗆From one year old to two years old

🗆It does not have a specific period and can be used at any time based on a physician's order.

1. Which of the following supplements is not common during pregnancy?

🗆Idofolic tablets

🗆Vitamin A + D drops or multivitamins

🗆Multivitamin tablets or capsules

🗆Iron tablets

🗆I do not know

1. Which of the following is not an important event in the "first 1000 days of life"?

🗆The development of a large part of the brain

🗆Learning to speak and gaining physical skills such as walking

🗆Laying the ground for the physical and mental health of adulthood

🗆The parturition and the birth of a baby

🗆Laying the ground for production and economic growth

1. How do you think people can achieve a more pleasant and happy life?

🗆By lowering their expectation levels and being contented

🗆Through constant work and effort to earn more income

🗆By creating a happy and loving environment in the family

🗆Happiness is relative and has no definite tactic.

🗆I do not know

1. Which option increases the likelihood of a child's academic and career success in adulthood the most?

🗆Fatherly love in childhood

🗆Motherly love in childhood

🗆Parental love in childhood

🗆Friend’s love in adolescent

🗆Spouse’ love after married

1. Which of the following roots the behavioral and psychological problems of a child?

🗆Violence and tension in the family during childhood

🗆Pregnancy stresses

🗆Hereditary background of the family

🗆Breastfeeding stresses

🗆All items

1. When is the best age to invest for the production and economic growth of society?

🗆Before any action to have children

🗆During pregnancy and early childhood

🗆During school

🗆During old aged

🗆All items

1. What are the root causes of diseases in adulthood?

🗆Internal and hereditary background – The events in the first 1000 days of life

🗆Internal and hereditary background – Environmental stimuli (stress, unhealthy food, polluted environment, etc.)

🗆Environmental stimuli (stress, unhealthy food, polluted environment, etc.) – The events in the first 1000 days of life

🗆Internal and hereditary background – The events in the first 1000 days of life – Environmental stimuli (stress, unhealthy food, polluted environment, etc.)

🗆Only the events in the first 1000 days of life

**Section3 (2 questions)**

1. Have you ever heard the phrase "the first 1000 days of life"?

🗆Very much

🗆Much

🗆Medium

🗆Little

🗆Not at all

2. If you have heard the phrase "the first 1000 days" or are familiar with it, please mention by which option you have got familiar with it.

🗆Cyberspace such as websites and social networks or social media such as radio and television

🗆Books and magazines

🗆Friends and relatives

🗆Healthcare staff

**Section4 (2 questions)**

1. How much do you like to know about the "first 1000 days of life"?

🗆Very much

🗆Much

🗆Medium

🗆Little

🗆Not at all

1. If you like to know; from what source do you like to get information?

🗆Mobile applications

🗆Media

🗆Books and Magazines

🗆Healthcare staff
